# Validation of the 3-item What Engagement Looks Like (WELL) scale in patients with diabetes

**DOI:** 10.1186/s41687-020-00225-6

**Published:** 2020-07-14

**Authors:** Suzanne Brodney, K. D. Valentine, Floyd J. Fowler, Michael J. Barry

**Affiliations:** 1 Informed Medical Decisions Program, Massachusetts General Hospital, Health Decisions Science Center, 100 Cambridge Street, 16th Floor, Boston, MA 02114 USA; 2 Health Decisions Science Center, Massachusetts General Hospital, 100 Cambridge Street, 16th Floor, Boston, MA 02114 USA; 3 Center for Survey Research, University of Massachusetts, Boston, 100 William T Morrissey Blvd, Boston, MA 02025 USA

**Keywords:** Patient engagement, Diabetes, Shared decision making, Measurement, Validation

## Abstract

**Background:**

Patients’ behaviors play a key role in chronic disease management, but how effective they are may depend on how engaged they feel. The objective was to develop a short measure of how much patients felt engaged in self-managing a chronic condition. Online test of a three-question series followed by a survey of physicians and their eligible diabetic patients. Physicians answered: 1) how well the physician thought the patient was managing his/her diabetes, and 2) how much effort the physician thought the patient was putting in. Each patient was mailed a survey that included three questions on self-management. Six hundred six patients from a national online consumer panel with diabetes or obesity, and 35 physicians from 3 primary care practices and a sample of 243 of their diabetic patients. Respondents were asked three questions about how much they thought their behavior could affect their health condition, how confident they were that they could do what was needed, and how involved they were in decisions about managing their condition. These items were summed to create a WELL score. Descriptive statistics and correlation coefficients were used to describe item relationships. Generalized Estimating Equations were used to predict how well the physician thought the patient was managing their diabetes and patient effort.

**Results:**

Correlations among the three patient-reported items ranged from − 0.01 to 0.45. The WELL score was correlated with an existing measure of patient activation commitment (*r* = .43, *p* < 0.001) and found to be a significant predictor of physicians’ ratings of how much effort patients devoted to condition management (b = 0.02, *p* = 0.001, OR = 1.02) after adjusting for confounders. The WELL score didn’t predict physicians’ ratings of how effective patients were (b = 0.003, *p* = .526, OR = 1.004) after their A1c score had been taken into account.

**Conclusion:**

Patients’ WELL scores predicted physicians’ ratings of patient effort in diabetes self-management.

## Background

Patients’ behaviors play a key role in chronic disease management, but how effective they are may depend on how engaged they feel in their own health care. However, there is not a one-to-one correlation between patient effort and effectively controlling a condition. For some patients, managing their chronic condition may require minimal effort to achieve optimal clinical outcomes. In other cases, patients may put forth significant effort, but are unable to achieve results that would be considered optimal. Clinicians commonly interact with patients aiming to improve the quality of their chronic condition management. Having better data on patients’ views of their self-management efforts may help strengthen the relationship between patients and clinicians and help clinicians identify patients in need of more intensive guidance and support.

Shared decision making (SDM) is a process of communication where clinicians and patients work together to make optimal healthcare decisions that align with what matters most to patients [1], and has been termed the “pinnacle of patient-centered care” [2]. SDM is used for making a ‘preference-sensitive’ decision, a decision when there is more than one reasonable option for how to treat that condition, and the best option depends on the patient’s goals and values related to that decision. Patients who engage in SDM supported by patient decision aids have better knowledge, participate more in decisions, and are less often undecided [3]. SDM may also be useful in chronic condition management, which can be seen as a series of decisions that rely on the patient’s ongoing involvement [4–9].

There is a growing body of evidence describing how to measure SDM and assess the quality of a preference-sensitive medical decision [10–13], but the majority of those studies focus on measuring SDM for major one-time decisions, such as surgical decisions, not ongoing decisions about how to manage chronic conditions. There are differences in how patients engage in SDM for a one-time decision compared to how they manage a chronic condition that includes initiating and sustaining a behavior change [14]. Several measures of how engaged or activated patients are have been studied across conditions associating higher patient activation with better health outcomes or behaviors [15–17]. The most widely used scales are relatively long, proprietary, and/or non-disease specific.

## Methods

The goal of this study was to develop and evaluate a brief, condition-specific, publicly available patient-reported outcome measure to assess patients’ self-report of how engaged they feel in managing a specific chronic condition. In this project, we chose to use obesity (in an initial study) and diabetes as the two target conditions for evaluating the measure.

This study was conducted in three phases: 1) cognitive interviews to understand how people managed their chronic health conditions, 2) an online survey of a national consumer panel to compare a new measure of patient engagement to an existing measure of patient activation, and 3) a validation study conducted in primary care clinics with patients and their primary care physicians (PCPs) to compare patients’ reports of self-management of their diabetes to the PCPs’ ratings of how well they thought the patients were managing their condition (Effort) and how effective (Effectiveness) they were.

### Qualitative testing

In 2015, a set of 10 questions were developed to better understand the extent to which people were engaged in managing their chronic health conditions and if these questions needed to be specific to a condition or could be asked more generally. To evaluate these questions, six cognitive interviews were conducted, three in person and three by phone to identify the key domains central to how patients interpreted the items. The participants ranged in age from 31 to 71 and 5 of the 6 had diabetes, 2 of 6 had chronic obstructive pulmonary disease, 1 of 6 had heart disease, 3 of 6 were high school graduates, and 4 of 6 were male. Based on these interviews, the research team, including survey methodologists and clinicians, identified three essential elements related to patients taking an active role (being constructively engaged) in managing their chronic conditions. The three constructs included: patients have to think being involved in managing their condition matters, they have to have confidence they can do what needs to be done, and they have to be involved in decisions about what they are going to try to do. The wording of the three questions was modified based on the qualitative feedback and focused on a specific condition. This preliminary work was not published. It was conducted by the Center for Survey Research at the University of Massachusetts at Boston (https://www.umb.edu/csr). The goal of this assessment was to provide qualitative data on how well people understood questions about managing their chronic conditions. 

### Online survey

In 2015, a sample of 606 participants was recruited by Survey Sampling International from their national online consumer panel to field test the three items and the response options (Table 1). Three hundred five of the respondents had been diagnosed with type 2 diabetes in the past year and 301 did not have diabetes but had been told by a health care provider in the past year that they should lose weight. The three items were included in the online survey along with the Short-Form Altarum Consumer Engagement Measure™ (SF-ACE) [17] (Table 1). A non-exclusive research use license agreement was obtained from Altarum Institute to use this scale in the research study. The SF-ACE is a 12-item instrument that measures three domains of health engagement: commitment, navigation and informed choice. We compared the three items from our qualitative testing to the commitment subscale of the SF-ACE, which was the most relevant subscale to what we were trying to measure.

**Table 1:** Surveys used in the online and primary care practices studies

Online survey		Primary care practices surveys	
Online test of WELL scale	SF-ACE commitment subscale	WELL scale	PCP questions on effectiveness and effort
Please rate ach of the following statements using any number from 0 to 5, where 0 is not at all and 5 is a lot. 1. How much do the things you do in your daily life affect your health? 2. How much confidence do you have in your ability to do things that will improve your health? 3. How much are you involved in decisions about how to manage your health?	Please rate how much you agree or disagree with the following statements: strongly disagree, disagree, neither agree nor disagree, agree, strongly agree. 1. I can stick with plans to exercise and eat a healthy diet. 2. Even when life is stressful, I know that I can continue to do the things that keep me healthy. 3. When I work to improve my health, I succeed. 4. I handle my health well.	Using any number from 0 to 10, where 0 is not at all and 10 is a lot. 1. How much do the things you do in your daily life affect your diabetes? 2. How much confidence do you have in your ability to do things that will help you manage your diabetes? 3. How much are you involved in decisions about how to manage your diabetes?	1. Using any number from 0 to 10 where 0 means not well at all and 10 means extremely well, what number would you give to how well this person is managing his or her diabetes now? 2. Using any number from 0 to 10 where 0 means not at all and 10 means a lot, how much effort is this person putting into doing the things he or she can reasonably do right now to manage his or her diabetes?

### Primary care practices

To test the validity of the scale we invited a convenience sample of primary care physicians (PCPs) from three primary care practices affiliated with Massachusetts General Hospital (MGH) to participate in a study. Two of these practices were community health centers, which serve a particularly diverse group of patients. Thirty-nine PCPs were invited to participate, and 35 opted in. The 35 PCPs were asked to review up to 15 patients from their ‘loyalty cohort’, a cohort of patients closely affiliated with a specific MGH PCP [18, 19]. The loyalty cohort appropriate for this study was identified as patients aged 18 or older, with at least three visits with the same PCP over the past 3 years, diabetes as a billing diagnosis, ability to read in English or Spanish, hemoglobin A1c level measured in the past 24 months, and currently a patient of the PCP. Since there was variation in the number of patients who met the eligibility criteria for each PCP, if a PCP had more than 15 patients meeting the inclusion criteria, 15 of their eligible patients were selected at random.

The PCP was sent a REDCap survey that included items for each of their (up to 15) patients. For each patient, the PCP confirmed eligibility, and if the patient wasn’t eligible, coded the reason. For those who were confirmed eligible, the PCP was asked whether the patient should receive an English or Spanish version of the paper survey. The final step in the survey was for the PCP to answer two questions about each patient: 1) how well the PCP thought the patient was managing his/her diabetes (Effectiveness), and 2) how much effort the PCP thought the patient was putting in to doing things he/she could to manage his/her diabetes (Effort) (Table 1).

Eligible patients were mailed a cover letter co-signed by their PCP, a fact sheet describing the study, a short survey, a postage-paid return envelope, and a small cash incentive. The Spanish versions of the documents were translated and certified. The patient received the mailed survey in English or Spanish, depending on what their PCP recommended. Study staff made up to three phone calls in English or Spanish to remind the patient about the survey followed by a reminder mailing and an additional phone call. The survey included the three items used in web survey with one exception: to increase the spread of answers respondents answered using a 0 to 10 scale rather than the 0 to 5 scale (Table 1). In addition, patients completed questions about overall health and education.

### Statistical analysis

#### Online survey

We hypothesized that the three items from our testing, which we refer to as the *What Engagement Looks Like (WELL) scale*, would be positively correlated with the SF-ACE commitment subscale. To counter any potential order effects from the online survey, half the study respondents were presented with the SF-ACE followed by the WELL scale. The order was reversed for the other half of respondents. We analyzed for differences between the two orders as well as differences between patients with diabetes or overweight using independent t-tests. Descriptive statistics were used to describe the online survey sample, as well as the 3-item WELL scale and the commitment subscale of the SF-ACE. To evaluate construct validity, we correlated the SF-ACE commitment subscale with the 3-item WELL scale.

To determine the appropriate sample size for the survey used in the primary care practices, the mean and standard deviation (SD) from the WELL scale in the online survey was calculated. The mean score on the WELL scale was about 18 with a SD of about 4.1. To detect a true difference of 0.5 SD between patients with lower versus higher scores on the PCP questions, with 80% power and a 2-sided alpha error of 0.05, a sample size of 64 per group, or 128 overall was required. To take into account the “nesting” of patients within PCPs in the analysis, we inflated the sample size 1.4 fold (assuming an ICC of ~ 0.1), or to 179 overall. Finally, assuming a 50% response rate, we anticipated approximately 358 eligible patients would need to be mailed a survey.

### Primary care practices

The primary hypothesis was that PCPs who rated their patients above the sample median on 1) how well the PCP thought their patient was managing their diabetes (Effectiveness) and 2) how much effort the PCP thought the patient was putting in to do the things he/she could to manage his/her diabetes (Effort) would have significantly higher WELL scores than those patients who scored below the median.

We examined the distribution of the WELL scale responses using the range, mean, standard error, skewness and top-score dichotomization. Each of the three questions had a response range of 0–10, and the range of the overall scale was 0–30. The relationships of the three items in the WELL scale were examined using Pearson correlation coefficients.

The distributions of responses for the two PCP questions are described and the correlation between the two PCP questions was calculated. A1c was dichotomized at < 7 and ≥ 7 to indicate whether the A1c was in control or not, respectively. Generalized Estimating Equations were used to predict each of the two questions the PCPs were asked, adjusting for A1c, language, overall health, age, race, education and sex. We also explored the relationship between the WELL scale and A1c, language, overall health, age, race, education and sex while adjusting for the nested nature of patients within PCPs.

## Results

### Online survey

Patients with diabetes and those who had been told to lose weight differed in their educational distribution and age, but not gender (Table 2).

**Table 2:** Patient characteristics for the online and primary care practices samples

	Online sample *N* = 606			Primary care practices sample *N* = 237
	Diabetes group	Overweight group	Overall	Overall
Characteristic				
Age, Mean (SD)	58.1 (11.9)	51.7 (14.0)	54.9 (13.4) Range (23–83)	66.7 (12.4) Range (20–93)
Women % (N)	52.8 (161)	56.15 (56.2) (169)	54.46 (54.5) (330)	52.7 (125)
Education % (N)				
Some high school or less	3.0 (9)	6.3 (4)	4.6 (13)	18.1 (43)
High school graduate/GED	33.8 (103)	60.5 (182)	47.0 (285)	24.5 (58)
Some college/2-year degree	26.9 (82)	18.3 (55)	22.6 (137)	25.7 (61)
4-year college degree	21.0 (64)	10.0 (30)	15.5 (94)	11.4 (27)
More than 4-year degree	15.4 (47)	5.0 (15)	10.2 (62)	20.3 (48)
Race^a^				
White, % (N)				70.0 (166)
Black, % (N)				7.6 (18)
Spanish-speaking^a^, % (N)				15.2 (36)
Overall health status^a^				
Fair/Poor, % (N)				40.9 (97)
Good, % (N)				40.1 (95)
Very good/excellent, % (N)				19.0 (45)
A1c^a^, Mean (SD)				7.2 (1.3)

^a^Data not collected for the online sample

Patients with diabetes tended to be older and had attained higher levels of education when compared to overweight patients (*p* < .001). The WELL scale scores for the combined group ranged from 0 to 30, with a mean of 22.05 (SD 4.9), skew of − 0.33 and 10.93% receiving the highest score. The range of the SF-ACE commitment subscale was 0–16, mean 9.96 (3.11), skew − 0.34, and 7.14% receiving the highest score. No difference was found between diabetic and overweight patients on the WELL scale (t (604) = − 0.917, *p* = 0.360, d = 0.07) or the SF-ACE commitment subscale (t (604) = 0.72, *p* = 0.472, d = 0.06). There was also no difference in the WELL scale or SF-ACE subscale based on the order of the scales in the survey (WELL t (604) = 1.54, *p* = .12, d = 0.13; SF-ACE subscale t (604) = 0.7, *p* = .67, d = 0.06). Correlations among the three WELL items ranged from 0.06 to 0.34 (Table 3). A 3-item composite WELL score was constructed by summing the responses. In the online survey, the WELL scale was significantly positively correlated with the SF-ACE commitment subscale, *r* = 0.43, *p* < .001.

**Table 3:** Correlation matrix for online and primary care clinic populations

Online sample				
	Daily life	Confidence	Decision management	WELL scale
Daily life	1			
Confidence	0.06 (0.14)			
Decision management	0.014 (< 0.01)	0.34 (< 0.01)	1	
WELL scale	0.68 (< 0.01)	0.68 (< 0.01)	0.65 (< 0.01)	1
Primary care population sample				
Daily life	1			
Confidence	−0.02 (0.77)	1		
Decision management	0.17 (0.01)	0.46 (< 0.01)	1	
WELL scale	0.65 (< 0.01)	0.63 (< 0.01)	0.77 (< 0.01)	1

*r* (*p*-value)

### Primary care practices

Of the 39 PCPs who agreed to participate, 35 completed an eligibility review of a total of 509 patients. Of the 121 categorized as not eligible, 12% were no longer patients of that PCP, 29% weren’t diabetic, 16% were unable to read English or Spanish, 11% were deceased, 9% had another illness precluding involvement, 8% had cognitive impairment/dementia and 16% did not meet other eligibility criteria. There was some overlap as PCPs could choose more than one exclusion category. After the PCP eligibility review, 388 of 509 initially eligible patients were sent a survey and 243 (63%) completed the survey. Six patients were removed because they did not complete the full WELL scale; this resulted in a final sample size of 237.

Differences between responders and non-responders on the two PCP items of interest were explored, adjusting for clustering by PCP and covariates. There were no differences between the groups except those in the main internal medicine clinic were more likely to be responders than those at the health centers (b = 0.80, *p* = .005, OR = 2.21).

Correlations among the three WELL items ranged from − 0.02 to 0.46 (Table 3). The item about being involved in decisions was not correlated with the answers to the other two items. A 3-item composite WELL score measure was constructed by summing responses.

Table 4 includes the range, mean, skew, percent top score and percent above median of the PCP rating questions on effort and effectiveness. The correlation between the two PCP questions was moderately high (*r* = 0.69, *p* < .001).

**Table 4:** Distribution of the WELL scale and PCP ratings

Measure	Range	Mean (SE)	Skew	Percent top-score	Percent above median when median split
WELL scale	0–30	20.1 (5.6)	− 0.68	3.1	Not applicable
PCP effort rating	1–10	6.4 (2.3)	− 0.46	7.2	52.7
PCP effective rating	0–10	6.6 (2.4)	−0.70	15.9	60.3

The WELL scale was found to be a significant predictor of physicians’ ratings of how much effort patients devoted to condition management (b = 0.02, *p* = 0.007, OR = 1.02) after adjusting for A1c, language, overall health, age, race, education and sex. For each point a patient increases on the WELL Scale, he/she was 2% more likely to be categorized as being above the median on the physician rating on effort. The WELL scale did not predict physicians’ ratings of how effective patients were (b = 0.003, *p* = 0.523, OR = 1.004) after adjusting for A1c, language, overall health, age, race, education and sex (Table 5). The patients’ A1c scores were the best predictors of physician ratings of effectiveness (b = 0.25, *p* < 0.001, OR = 1.28).

**Table 5:** Predicting physician ratings of effort and effectiveness

Predicting physician ratings of effort				Predicting physician ratings of effectiveness			
Predictor	b (SE)	p	OR	Predictor	b (SE)	p	OR
Intercept	−0.36 (0.25)	0.15	0.7	Intercept	−0.19 (0.24)	0.42	0.82
WELL Scale	0.02 (0.01)	< 0.01	1.02	WELL Scale	0 (0.01)	0.52	1
A1C in Control	0 (0.07)	0.95	1	A1C in Control	0.25 (0.06)	0	1.28
Language (Spanish)	−0.02 (0.11)	0.82	0.98	Language (Spanish)	−0.03 (0.11)	0.79	0.97
Overall Health	0.11 (0.04)	< 0.01	1.12	Overall Health	0.11 (0.04)	< 0.01	1.12
Age	0 (0)	0.14	1	Age	0 (0)	0.25	1
Race (White)	−0.12 (0.08)	0.16	0.89	Race (White)	−0.08 (0.08)	0.35	0.93
Education	−0.01 (0.02)	0.77	0.99	Education	0.03 (0.02)	0.23	1.03
Gender (Male)	0.06 (0.06)	0.34	1.06	Gender (Male)	0.09 (0.06)	0.15	1.09

Although the WELL score was not independently associated with whether or not A1c was in control (*r* = − 0.05, *p* = .47), we found that when we adjusted for other variables (such as age, race, language, sex, education, and overall health) and accounted for variability due to the physician, these measures were related (b = − 1.45, *p* = .051). This finding indicated that overall, those whose A1c was not under control had higher WELL scores.

## Discussion

The objective of this project was to develop a short, publicly available measure of patient engagement that could help PCPs better understand how patients felt they were managing their chronic condition. After qualitative testing to refine the questions, we assessed the construct validity by comparing our measure to the SF-ACE commitment subscale, a validated measure of patient activation. To test that our measure was capturing how engaged patients were, we tested the survey with 35 PCPs and 237 of their patients with diabetes. We asked PCP to rate how effective each of their patients was at managing their condition and how much effort the PCP thought the patient was putting in. We found that after controlling for covariates, the patient-reported WELL Scale predicted physicians’ ratings of the patients’ effort, but not how effective patients were at managing their conditions. A1c, on the other hand, was the most significant predictor of how effective the physician thought the patient was at managing their diabetes. Patients with A1c ≥7 were more likely than average to report higher WELL scores, suggesting they were more engaged in working on their diabetes.

Chronic condition management includes a series of decisions over time between the patient and provider, which has been called the Shared Treatment Decision-Making approach in chronic disease [5]. This approach is based on a partnership between the patient and provider, information exchange, deliberating on options and deciding and acting on a decision. However, there are challenges to measuring if SDM has occurred in chronic condition management since typical measures have focused on a ‘preference-sensitive’ rather than the ongoing involvement in daily decisions. There is a need for a measure that could be used in clinical practice to give clinicians feedback on how engaged patients are, and to identify those who need additional support.

There are several limitations to our research. This online survey identified patients who were self-reporting their condition. Demographics descriptors of the online sample were not collected so this limits the ability to generalize the findings. Overall, we do not know from our data if patients who are more engaged as measured by the WELL scale will be more successful over time than those who are categorized as less engaged in controlling their diabetes.

Future research will include exploring what activities patients with higher WELL scores are doing to manage their condition, such as changes to their diet, weight or physical activity level. In addition, we will focus on establishing reliability and validity in patients with other chronic conditions.

## Conclusion

We developed a brief, valid measure that is publicly available to measure how engaged patients are in managing their diabetes. Patients’ WELL scores predicted physicians’ ratings of patient effort in diabetes self-management and preliminarily appears to be a promising measure of patient engagement in condition management. The WELL score indicates that those who are less in control of their diabetes tend to be working the hardest to improve. Patients’ behaviors play a key role in chronic disease management, but how effective they are may depend on how engaged patients feel. This measure of patient engagement could help clinicians better understand how patients are working to manage their chronic conditions.

## Data Availability

The datasets used and/or analyzed during the current study are available from the corresponding author on reasonable request.

## Abbreviations

WELL: What Engagement Looks Like
PCP: Primary care physician
SDM: Shared decision making
SF-ACE: Short Form Altarum Consumer Engagement
MGH: Massachusetts General Hospital
